# Detection and quantification of Lyme spirochetes using sensitive and specific molecular beacon probes

**DOI:** 10.1186/1471-2180-9-43

**Published:** 2009-02-24

**Authors:** Diana S Saidac, Salvatore AE Marras, Nikhat Parveen

**Affiliations:** 1 Department of Microbiology and Molecular Genetics, University of Medicine and Dentistry of New Jersey, New Jersey Medical School, Newark, NJ 07103-3535, USA; 2 Public Health Research Institute and Department of Microbiology and Molecular Genetics, University of Medicine and Dentistry of New Jersey, New Jersey Medical School, Newark, NJ 07103-3535, USA

## Abstract

**Background:**

Lyme disease, caused by *Borrelia burgdorferi*, affects a large number of people in both the USA and Europe. The mouse is a natural host for this spirochete and is widely used as a model system to study Lyme pathogenesis mechanisms. Since disease manifestations often depend upon the spirochete burden in a particular tissue, it is critical to accurately measure the bacterial number in infected tissues. The current methods either lack sensitivity and specificity (SYBR Green), or require independent analysis of samples in parallel to quantitate host and bacterial DNA (TaqMan). We have developed a novel molecular beacon-based convenient multiplex real-time quantitative PCR assay to identify and detect small numbers of *B. burgdorferi *in infected mouse tissues.

**Results:**

We show here that molecular beacons are effective, sensitive and specific probes for detecting and estimating wide-ranging numbers of *B. burgdorferi *in the presence of mouse DNA. In our assays, the spirochete *recA *and the mouse *nidogen *gene amplicons were detected simultaneously using molecular beacons labeled with different fluorophores. We further validated the application of these probes by quantifying the wild-type strain and *bgp*-defective mutant of *B. burgdorferi*. The *bgp*-defective mutant shows a ten-fold reduction in the level of spirochetes present in various tissues.

**Conclusion:**

The high sensitivity and specificity of molecular beacons makes them superior probes for the detection of small numbers of *B. burgdorferi*. Furthermore, the use of molecular beacons can be expanded for the simultaneous detection and quantification of multiple pathogens in the infected hosts, including humans, and in the arthropod vectors.

## Background

Lyme disease, caused by the spirochete *Borrelia burgdorferi*, is a highly prevalent multisystemic illness that affects the heart, joints, skin, musculoskeletal and nervous system. Persistent infection with the spirochete results in potentially severe manifestations, such as, carditis, arthritis, acrodermatitis chronicum atrophicans and neuroborreliosis. The severity of the Lyme disease depends on several factors including; genotypes of both the host and the infecting Borrelia strain, age of the host, simultaneous infection with another tick-transmitted pathogen and the spirochete burden in the infected tissue [1-7].

The *B. burgdorferi *genome is relatively small (1.52 Mb) in size. Although the spirochete lacks major biosynthetic pathways, it contains a large number of surface proteins. Several of these are adhesins, which mediate attachment to various cell lines [8-13]. Each adhesin could contribute to the tissue specific colonization by the spirochetes. Alternatively, the presence of multiple adhesins exhibiting specificity for the same receptor can create a redundancy of function [9,14]. In the latter case, a mutation in the gene encoding a particular *B. burgdorferi *adhesin can only moderately reduce the ability of the spirochete to colonize. Indeed, mutation in a specific spirochete gene has been shown to reduce the number of *B. burgdorferi *in the infected tissues [15,16]. Therefore, although Bgp is not essential for infection it could contribute to tissue colonization by Lyme spirochetes. A sensitive detection system is critical to assess the burden of these mutant spirochetes in tissues and to determine the impact of mutation on a specific disease manifestation, and hence, could provide insight into the role of unique genes of *B. burgdorferi *in Lyme disease. Quantification of the spirochete burden in infected tissues by Real-time quantitative PCR (qPCR) using the fluorescent dye, SYBR Green I, is a commonly used method [5,6,17,18]. However, this dye binds to the minor groove of the DNA double helix in a sequence-independent manner. Therefore, it is susceptible to detection of non-specific amplification products, including primer dimers.

Several types of fluorogenic hybridization probes have been described for the specific detection of PCR amplified products. The best characterized among these are the TaqMan probes. These probes are single stranded oligonucleotides labeled with a fluorophore-quencher pair that hybridize with the sequence present in the internal region of an amplified PCR product. When free in solution, TaqMan probes form random coils to bring fluorophore reporter and quencher in close proximity, enabling Fluorescence Resonance Energy Transfer (FRET) from the fluorophore to the quencher. This mechanism alleviates the fluorescence signal of the reporter. In the presence of the target, the TaqMan probe-target hybrid comes in contact with the Taq Polymerase during the extension phase of a PCR cycle. The inherent 5'exonuclease activity of the enzyme then cleaves the probe, releasing the fluorescent reporter from the probe. This prevents FRET and leads to an increase in the fluorescence intensity at each subsequent PCR cycle. Several researchers have employed this technique effectively to quantify *B. burgdorferi *in mammalian tissues and in ticks [15,16,19-26]. However, simultaneous quantification of spirochete and infected mammalian DNA has not been described.

The proximity of the fluorophore and the quencher in TaqMan probes in the free state depends on the formation of random coils and often results in only partial quenching of the fluorescence, and hence, can produce a high background [27]. In contrast, molecular beacon probes are single-stranded oligonucleotides that form stem-loop structures with the recognition sequence mainly located in the loop region. A 5–7 base pair stem brings the fluorophore at the 5'end and non-fluorescent quencher at the 3'end together [28]. This contact-dependent quenching mechanism is highly efficient and reduces the background fluorescence significantly when the probe is free in solution. The presence of the target sequence leads to the formation of a probe-target hybrid, which is longer and more stable than the stem. This spontaneous conformational reorganization forces dissociation of the fluorophore and the quencher resulting in a significant increase in fluorescence. Because of the specificity of the interaction between the probe region of the molecular beacon with the complementary target sequence within the PCR amplification product, the presence of the non-specific DNA does not interfere with the quantitative detection of the intended amplification product.

Due to their potential superiority [27], we used molecular beacons for PCR-based quantification of *B. burgdorferi *in this study and assessed their efficiency, sensitivity and specificity relative to the SYBR Green I based detection system. Furthermore, the molecular beacons were used to detect *B. burgdorferi*, including the *bgp *mutant, in infected mouse tissues effectively.

## Results

### Analysis of molecular beacon probes for qPCR detection of *recA *gene of *B. burgdorferi *and *nidogen *gene of mouse

**Figure 1:**
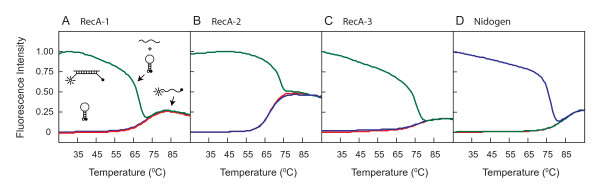
**Melting curves of RecA and Nidogen molecular beacon probes in the presence of specific or unrelated targets**. Melting curves between the RecA1, RecA2 and RecA3 molecular beacons (A-C) in the presence of complementary target sequences (green lines), in the presence of unrelated Nidogen target sequence (blue lines) or in the absence of any target (buffer only control, red lines) were generated. The fluorescence analyses indicate that the molecular beacons exist either as hybrids with their targets, exhibiting high fluorescence or are in the free state in the form of a stem-loop structure with fluorescence quenched at a temperature range of 55–75°C. A similar analysis of a Nidogen molecular beacon depicted a temperature and fluorescence profile (D), which is similar to the RecA3 molecular beacon.

The specificity of each molecular beacon for its respective amplicon was first determined by generating the denaturation profiles for each of three RecA probes with specific or irrelevant target oligonucleotides (Table 1; Figure 1). In the presence of the unrelated Nidogen target or in the absence of any target (buffer control), RecA1, RecA2, and RecA3 molecular beacons remain in a closed state at low temperatures with fluorophore and quencher held in close proximity by the hairpin formation. Molecular beacons remain dark at this state (1A, 1B and 1C). At temperature above the melting temperatures of the stems (71°C, 67°C and 75°C for RecA1, RecA2 and RecA3, respectively), the fluorophore separates from the quencher resulting in increase in fluorescence intensity. In contrast, these molecular beacons bind to their respective targets at low temperature resulting in the dissociation of the stem and an increase in fluorescence. At the melting temperatures of probe-target hybrids (68°C, 73°C and 75°C for RecA1, RecA2 and RecA3, respectively), dissociation of the probe from the target results in the return of the probe to a stem-loop structure, significantly diminishing the fluorescence. On further increase in temperature, the beacons denature completely, do not form a stem-loop structure, and hence, start to emit fluorescence. Cartoons in the figure depict different molecular beacon states at particular temperatures, in the presence or absence of specific targets in the reaction. Although the denaturation profiles of RecA1 and RecA3 seem similar, only RecA3 showed high fluorescence signal for detection of *B. burgdorferi *in the presence of mouse DNA by qPCR.

**Table 1:** Sequence of primers for PCR, molecular beacon probes and their specific targets

PCR Primers, Probes and Targets	Sequence	Length	Fluorophore/Quencher	Tm Probe-target/Stem
RecF	5' GTG GAT CTA TTG TAT TAG ATG AGG CTC TCG 3'	30	-	-
RecR	5' GCC AAA GTT CTG CAA CAT TAA CAC CTA AAG 3'	30	-	-
NidoF	5' CCA GCC ACA GAA TAC CAT CC 3'	20	-	-
NidoR	5' GGA CAT ACT CTG CTG CCA TC 3'	20	-	-
Nidogen	5' CGG CGC**ACC CAG CTT CGG CTC AGT** AGC GCC G 3'	31	TET/BHQ1	77°C/84°C
Nidogen Target	5' ta GGC GCT ACT GAG CCG AAG CTG GGT G at 3'	29	-	-
RecA1	5' CCC GCG**CGT CTG GCA AGA CTA CTT TAA CTC** TTC GCG GG 3'	38	FAM/BHQ1	68°C/71°C
RecA1 Target	5' ta GAA GAG TTA AAG TAG TCT TGC CAG ACG at 3'	31	-	-
RecA2	5' CGCGAG**TCG TCT GGC AAG ACT ACT TTA A**CTCGCG 3'	34	FAM/DABCYL	73°C/67°C
RecA2 Target	5' ttG AGT TAA AGT AGT CTT GCC AGA CGA CTC tt 3'	32	-	-
RecA3	5' CTG GCG**GAT ATC CTA GGG GG**CGC CAG 3'	26	FAM/BHQ1	75°C/75°C
RecA3 Target	5' ttG CGC CCC CTA GGA TAT CCG CCt t 3'	25	-	-

Underlined letters in molecular beacons sequence indicate stem sequence and bold letters indicate the probe (loop) sequence. There is an overlaps between probe and stem sequence in RecA3 molecular beacon.

Nucleotides denoted by small case letters in the targets indicate non-template based tails

FAM-Fluorescein, TET-Tetrachlorofluorescein, DABCYL-[4 - ((4 - (dimethylamino)phenyl)azo) benzoic acid] and BHQ-1 = Black Hole Quencher 1

**Figure 2:**
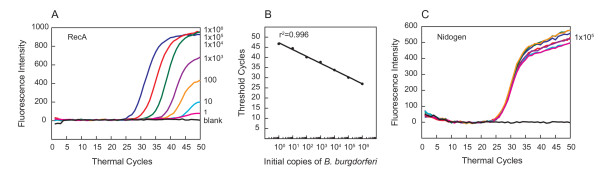
**Molecular beacons can detect *B. burgdorferi *between 1 and 10^6 ^in multiplex assay, when C3H mouse DNA was also included**. Amplification plots of *recA *and *nidogen *genes in PCR assays to estimate quantities of *B. burgdorferi *(A) and mouse (C) DNA are shown. Uninfected mouse heart DNA (containing 10^5 ^*nidogen *copies) spiked with ten-fold dilutions of *B. burgdorferi *strain N40 ranging from 1 to 10^6 ^were used in the PCR assays containing both RecA3 and Nidogen molecular beacons. Sensitivity and specificity of the detection system is indicated by the ability of RecA3 and Nidogen molecular beacons to quantify the amplicons from both the *recA *and the *nidogen *genes in the same PCR assay tubes. A high coefficient of correlation (r^2 ^= 0.996) between the Ct values and the spirochete number obtained from the standard curve (B) indicates that the molecular beacons can be used effectively to quantify spirochete burden in infected tissues using multiplex assay system.

Similar denaturation profiles generated with the Nidogen molecular beacon in the presence of (1) the complementary sequence target, (2) unrelated RecA target, or (3) the buffer alone indicated similar fluorescence profiles (Figure 1D). Melting temperatures of Nidogen and RecA3 probe-target hybrids are comparable (77°C and 75°C respectively) due to the high GC content of the probes. In addition, highest detection sensitivity for *B. burgdorferi *was obtained using the RecA3 molecular beacon (Figures 2, and data not shown). Therefore, we used the RecA3 molecular beacon for all further experiments.

### *B. burgdorferi *and mouse DNA can be quantified simultaneously using molecular beacons in multiplex system

Since molecular beacons are specific hybridization probes for particular PCR products, simultaneous detection of pathogen and host PCR products is possible using molecular beacons tagged with different fluorophores. Therefore, normalization of the host DNA in different tissue samples is more convenient and accurate. To test this premise, a ten-fold serial dilution of genomic DNA of *B. burgdorferi *strain N40 spiked in the same concentration of the uninfected mouse tissue DNA, i.e., 10^5 ^*nidogen *copies per reaction, were used as template for the PCR assays. The "threshold cycle" (Ct) is the PCR cycle at which specific fluorescence rises significantly above the fluorescence background. In this assay, the threshold was set at twenty times the standard deviation of the noise in the background fluorescence of each PCR assay (recorded between the third and 20th thermal cycle).

**Figure 3:**
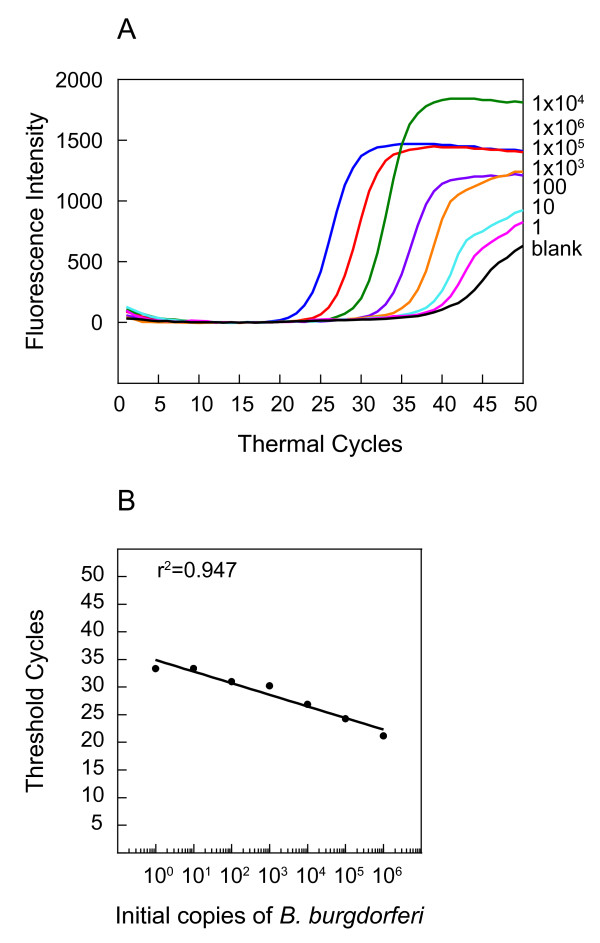
**SYBR Green 1, a non-specific double stranded nucleotide fluorescent probe, can detect a wide range of *B. burgdorferi *numbers in the presence of mouse DNA**. The amplification plots (A) show PCR of the *recA *gene of *B. burgdorferi *strain N40 as detected by SYBR Green at the end of each PCR cycle. Uninfected mouse joint DNA (containing 10^5 ^nidogen copies) spiked with a ten-fold dilution of *B. burgdorferi *DNA, starting with 10^6 ^spirochete copies, was used for this assay. A standard curve (B) and a direct correlation (r^2 ^= 0.947) between Ct number and *B. burgdorferi *number shows that a wide range of spirochete numbers can be detected in our system using SYBR Green.

Amplification plots of the *recA *gene in the PCR assays (Figure 2A), as detected by fluorescence intensity at the end of each cycle, show that the presence of 1 to 10^6 ^spirochetes can be detected using the RecA3 molecular beacon. Indeed, presence of ten spirochetes in a reaction was detected consistently in different assays, indicating reproducibility and sensitivity of this detection probe (data not shown). However, presence of approximately one spirochete in the reaction mixture was sometimes indistinguishable from background noise. A standard curve (Figure 2B) generated by plotting the log of the known initial copy numbers of *B. burgdorferi *versus the Ct values indicates that the threshold cycle is inversely proportional to the number of target molecules present in the samples. A high coefficient of correlation (r^2 ^= 0.996) between the *B. burgdorferi *copy number and the threshold cycle number (Ct) obtained from the standard curve indicates that this curve can be used to determine the quantity of spirochetes in infected mouse tissues. Furthermore, identical Ct values for *nidogen *in all samples indicate that the number of copies of *B. burgdorferi *genome in the sample does not interfere with the amplification and detection of the *nidogen *in the PCR assays (Figure 2C). This further confirmed the effectiveness and sensitivity of molecular beacons in multiplex analyses. SYBR Green I dye was used as a control in the PCR assays conducted in parallel using aliquots from the same serially diluted *B. burgdorferi *samples with *recA *primers (Figure 3A) as used above for generating the figure 2A. Although a direct correlation (r^2 ^= 0.947) between the spirochete copy numbers and Ct values was also observed using SYBR Green I (Figure 3B), an accurate spirochete burden was not detected reproducibly when the *B. burgdorferi *counts were ten or fewer in the sample. Lower sensitivity of the detection by SYBR Green 1 has also been a concern of other researchers [5,6,17,18].

**Figure 4:**
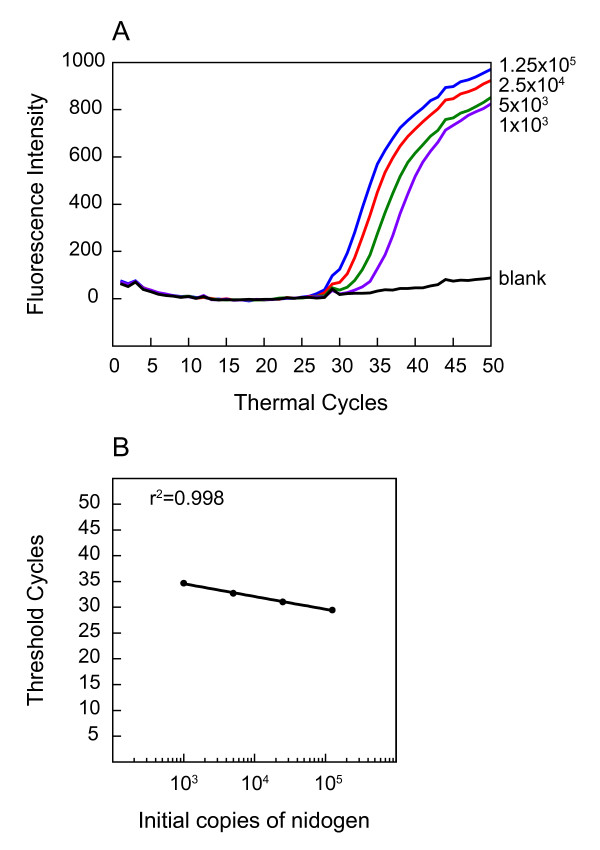
**A serial dilution of mouse joint DNA is detectable by Nidogen molecular beacons**. Amplification plots of five-fold dilution of mouse DNA used in PCR assays with Nidogen molecular beacon for detection of *nidogen *amplification products are shown (A). A standard curve (B) and high coefficient of correlation (r^2 ^= 0.998) indicates that the Nidogen molecular beacon is effective in detecting 200 ng to the low level (1 ng) of mouse DNA.

We further examined whether the detection of *B. burgdorferi *by molecular beacons is affected by the kind of mouse tissue used. A comparison of different dilutions of the spirochetes in C3H/HeN mice DNA (10^5 ^*nidogen *copies/reaction) from joints, skin and heart did not show significant variation in Ct values (Figures 2, 4, and data not shown). Therefore, quantification of *B. burgdorferi *in different tissues of infected mice is feasible using the same standard curve (Figure 2B). We also prepared a five-fold dilution of the uninfected mouse genomic DNA, starting with 10^5 ^*nidogen *copies for PCR, using a Nidogen molecular beacon probe. Amplification plots (Figure 4A) and the standard curve between mouse *nidogen *gene copy number and respective Ct values (Figure 4B) indicate that low number of *nidogen *copies, up to those obtained from 1ng DNA, can be detected by specific molecular beacons. A high coefficient of correlation (r^2 ^= 0.998) indicates that the quantity of the infected mouse tissue DNA can also be estimated from the Ct values obtained in a multiplex analysis. Further increase in mouse genomic DNA concentration (more than 200 ng DNA or >10^5 ^copies of *nidogen *in the reaction) hampered the PCR amplification. Therefore, to maintain sensitivity of the assay and accurate quantification of *B. burgdorferi *in the infected tissues using molecular beacons, the samples should be diluted to obtain 200 ng or less total DNA per reaction.

### Sensitivity and specificity of detection of qPCR amplicons is not affected by multiplex analysis

**Figure 5:**
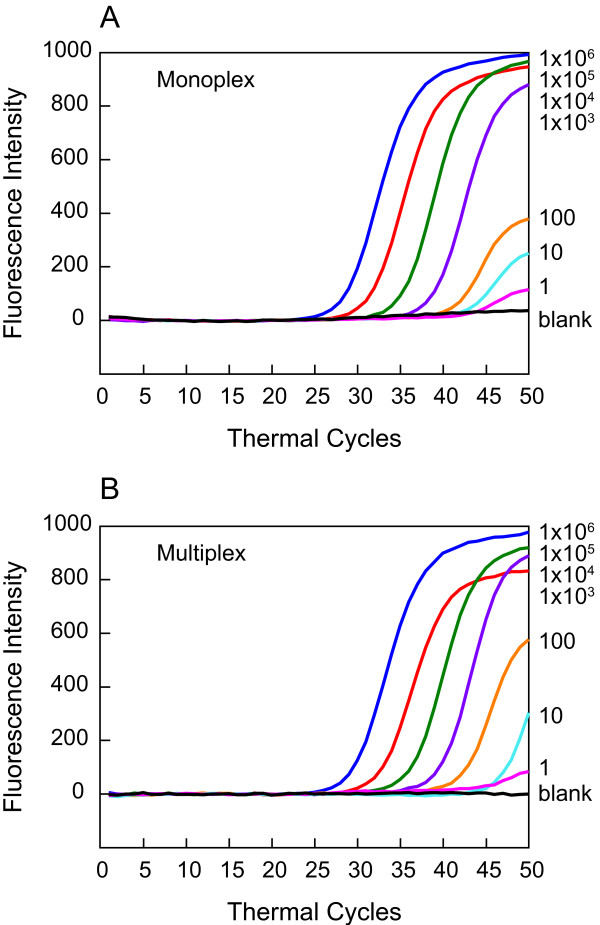
**Multiplex analysis does not affect sensitivity of detection of *B. burgdorferi *by molecular beacons**. A comparison of monoplex (A) and multiplex (B) assay systems of different dilutions of *B. burgdorferi *spiked in the mouse DNA containing 10^5 ^*nidogen *copies indicates that multiplex analysis does not affect the sensitivity of spirochete detection.

Quantity of *B. burgdorferi *in the infected tissues has been determined using conventional monoplex assays in which spirochete-specific primers and detection reagent (SYBR Green dye or TaqMan probe) are incorporated in the qPCR assay. This quantification involves simultaneous isolation of host and pathogen DNA. Therefore, the sensitivity of the detection of the spirochetes could be affected in multiplex analyses. Molecular beacons can simultaneously detect more than one amplicon, i.e., both the pathogen and the host, in the same reaction tube. To examine if sensitivity of detection by molecular beacons diminishes in multiplex analyses, a comparative analysis of the serially diluted *B. burgdorferi *in the mouse tissues was conducted in monoplex and multiplex assay systems. Uninfected C3H mouse tissue DNA (10^5 ^*nidogen *copies) was spiked with DNA from 10^6^*B. burgdorferi *followed by ten-fold dilution in same concentration of mouse DNA. Both set of primers, for *recA *and *nidogen *amplification, were added in each reaction. Only one molecular beacon was used at a time for monoplex assays while both RecA3 and Nidogen molecular beacons were included in multiplex assays. Sensitivity of detection of *B. burgdorferi *was high both in monoplex (Figure 5A) and multiplex assays (Figure 5B). Although a slight delay in Ct values was observed in multiplex relative to monoplex system (Figure 5), both monoplex and multiplex analyses show good correlation and are able to detect as little as one copy number of *B. burgdorferi*. Hence, the presence of primers and a molecular beacon for *nidogen *amplicon does not affect sensitivity of detection of *B. burgdorferi*. Thus, a multiplex assay system can be employed to accurately quantify Lyme spirochetes in infected mammalian tissues.

### Molecular beacons can be used effectively to quantify *B. burgdorferi *in the infected tissues

**Figure 6:**
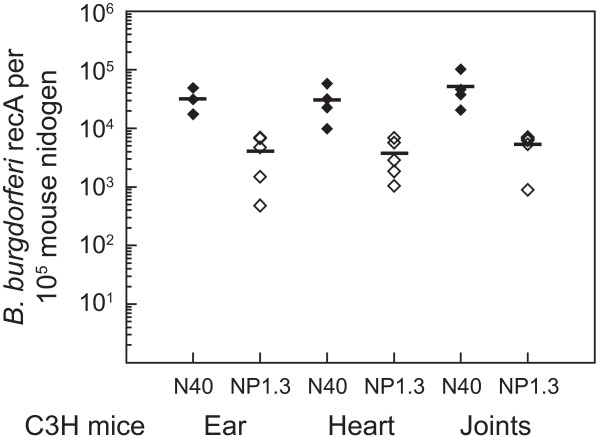
**Multiplex analysis of mouse infected tissues using molecular beacons indicate that *bgp*-defective mutant, NP1.3, is less efficient in tissue colonization than the wild-type N40 strain**. Number of *B. burgdorferi *strain N40 (filled diamonds) or NP1.3 (open diamonds) present in different tissues at two weeks of infection of C3H/HeN mice were determined by qPCR using molecular beacons. The spirochete load was normalized to 10^5 ^*nidogen *copies. After determination of the Ct values for *recA *of *B. burgdorferi *and mouse *nidogen *in the PCR assays, the standard curve (Figure 2B) was used to determine the number of spirochetes per 10^5 ^*nidogen *copies (~6 × 10^4 ^cells) of the infected mouse tissues.

To determine the applicability of the molecular probes in quantification of *B. burgdorferi *burden in the infected tissues, multiplex qPCR was conducted for ear, heart and joints of C3H/HeN mice infected either with N40 or its *bg*p-defective mutant, NP1.3. Since live NP1.3 mutants from tissues could not be recovered consistently by culture when infection dose was 5000 spirochetes per mouse (data not shown), an infection dose of 5 × 10^4 ^spirochetes per animal was used in this experiment. The Ct values for spirochetes were normalized for 10^5 ^copies of the mouse *nidogen *gene in each PCR, using the standard curve (Figure 2B). The results indicate that even though the NP1.3 strain can colonize the heart, joints and ear, the average burden of these mutant spirochetes in all tissues was approximately ten fold lower than that of the wild-type N40 strain (Figure 6).

## Discussion

Quantitative PCR is a widely used method for determining the burden of pathogens, including the Lyme disease-causing spirochetes, present in infected tissues. The fluorescent dye SYBR Green I, which binds non-specifically to double stranded DNA, has mainly been used to detect the qPCR product obtained for the *recA *or *fla *genes of *B. burgdorferi *for quantification. However, sensitivity of this detection system is poor when the number of spirochetes present in the tissues is low [8,29]. To overcome the background fluorescence obtained by binding of SYBR Green to the non-specific amplified products, such as primer dimers [17], a higher temperature (80°C) is needed for the detection of the amplicon. This could also contribute to the low sensitivity of this detection system when a small spirochete population and high primer dimer concentrations are present. Clinical Lyme disease manifestations are not always dependent on high *B. burgdorferi *burden. Furthermore, qPCR of a mouse gene, such as *nidogen*, using specific primers needs to be conducted separately to normalize the quantity of mouse tissue in the sample when SYBR Green is used. Hence, it is important to explore newer, more specific probes, which remain sensitive even when less than one hundred spirochetes are present in the PCR sample.

More recently, TaqMan probes have also been employed for detection of the PCR products from both *B. burgdorferi *and host/vector genes [16,19-26]. Although TaqMan probes have been reported to be a sensitive detection system for PCR of *B. burgdorferi *amplicon by several laboratories [19-22,24,25], high background fluorescence of the unhybridized probe, i.e., low signal-to-noise ratio, and lower sensitivity due to incomplete enzymatic hydrolysis has been observed with these probes [19,20,27]. In addition, compatibility of the fluorophore and quencher due to the requirement for sufficient spectral overlap remains a significant issue due to the requirement of FRET in TaqMan probes. This limits its application in the multiplex analysis to some extent. To the best of our knowledge, simultaneous detection of mouse and spirochete DNA using TaqMan probes in multiplex analysis has not been reported. In contrast to TaqMan probes, quenching due to a direct interaction between fluorophore and quencher in molecular beacons is much more efficient. It also offers a choice of a variety of fluorophores with quenchers. Indeed, the efficiency of molecular beacons is not affected significantly by the choice of different fluorophores-quencher combinations [30]

Denaturation profiles of the Nidogen molecular probe as well as three different RecA molecular beacons, and detection of *B. burgdorferi *by PCR assays indicate that RecA3 emits most fluorescence and shows the highest sensitivity of detection. RecA3 has a high GC content, and thereby, forms the most stable probe-target hybrid and hairpin structures. Furthermore, its detection step temperature is most compatible with that of the Nidogen molecular beacon (Table 1). This also makes RecA3 most suitable for multiplex analyses. The ABI7700 sequence detector software from Applied Biosystems can distinguish the emission of a particular fluorescence signal (from FAM or TET fluorophores) associated with each molecular beacon in PCR assays. Lower background signal facilitated the efficient detection of *B. burgdorferi *at seven different dilutions, and a high co-efficient of correlation between Ct values and spirochete number (r^2 ^= 0.996) was obtained. In addition, sensitivity of detection of *B. burgdorferi *DNA was not affected by the presence of mouse DNA and remained comparable in monoplex versus multiplex analyses. These results, as well as a high correlation (R^2 ^= 0.998) between threshold cycle number and the amount of mouse DNA, made quantification of the spirochetes burden in different infected mouse tissues convenient and accurate since a single PCR tube per sample was used for the analysis of both *B. burgdorferi *and mouse amplicons. This could be of great importance if this system is employed for detection of *B. burgdorferi*, as well as other pathogens, in patient tissues or fluids, where quantities of samples are often limiting.

Efficient amplification by PCR due to the small size of the amplicons and high signal to noise ratio obtained by the use of molecular beacon probes resulted in high sensitivity of this assay. However, a strong TET signal from the Nidogen molecular beacon sometimes hampered the sensitivity of detection of approximately one spirochete in the sample in multiplex systems (unpublished observation). This can be overcome by synthesizing molecular beacons with a combination of red (such as Texas red) and green (TET or FAM) fluorophore for use in multiplex analyses. This will be especially useful when the pathogen is present in very small numbers in the infected tissues.

Simultaneous infection by several pathogens often creates difficulty in identifying the causative agent for a particular disease manifestation. Multiplex analysis using molecular beacons allows detection of a pathogen and the host tissue by PCR. Furthermore, additional pathogen(s) can be detected by including the appropriate molecular beacon in the assay. This is possible for up to seven molecular beacons, each labeled with different fluorophores, which can be combined in one reaction to detect different amplicons, as long as PCR conditions are compatible. This is of great importance especially for the detection of multiple vector-borne bacterial illnesses in humans such as Lyme disease and human granulocytic anaplasmosis (HGA), caused by *Anaplasma phagocytophila*. Both of these organisms, along with several viruses, can be transmitted together to humans by *Ixodes *ticks, often complicating the diagnosis of Lyme disease. This study is focused on quantification specifically of *B. burgdorferi*, and not other Lyme spirochetes, in the mouse tissues. We anticipate that in the future, after slight modifications of the primers and molecular beacon, we will be able to distinguish the presence of different Lyme spirochetes in clinical samples. An appropriate human gene region will also be selected for amplification and a specific molecular beacon designed for diagnostic purposes. In addition, we will be able to detect Lyme spirochetes in combination with other organisms in clinical samples after an infected tick bite using the specific primers and different fluorophore-tagged molecular beacons. This will help to identify the actual causative agent, facilitate proper treatment strategy and offer a better clinical outcome for the patient. Furthermore, it will be possible to adapt this system to detect microbes in other systems, such as in the infected plants.

## Conclusion

In conclusion, molecular beacons have several advantages over other fluorescent probes for qPCR or Real-Time PCR including; (1) specificity of interaction with a particular amplicon, (2) possibility to select a variety of compatible fluorophores and quenchers, which show minimum interference, (3) high signal-to-noise ratio resulting in sensitivity of detection of the amplicons at low copy number, and (4) multiplex analysis (potentially of up to seven different amplicons simultaneously), which may make it feasible to detect multiple pathogens in infected tissue. Hence, molecular beacon probes will be very useful for the detection of various microbial pathogens in patients in the future.

## Methods

### Bacterial strains and mouse infection

N40, clone D10/E9, is an infectious *B. burgdorferi *(sensu stricto) isolate. We generated *bgp*-defective mutant of this strain, NP1.3, by disruption of the gene with a kanamycin resistance cassette [14]. Both *B. burgdorferi *strains were grown at 33°C in BSKII medium containing 6% rabbit serum. To conduct the infection studies, immunocompetent C3H/HeN mice were injected subcutaneously at a dose of 5 × 10^4 ^spirochetes per mouse. Mice were euthanized after two weeks of infection and tissues harvested for qPCR. UMDNJ-New Jersey Medical School is accredited (Accreditation number 000534) by the International Association for Assessment and Accreditation of Laboratory Animals Care (AAALAC International), and the animal protocol used was approved by the Institutional Animal Care and Use Committee (IACUC) at UMDNJ.

### Purification of *B. burgdorferi *and mouse genomic DNA

Total genomic DNA was isolated from the low passage *B. burgdorferi *strain N40 grown to a density of 10^8^spirochetes/ml using the protocol we described previously [10]. DNA from mouse tissues was isolated using the previously described protocol [17] with two modifications. Firstly, PLG-containing tubes (Qiagen Sciences, MD) were used for phenol and chloroform extraction, since they allow clean separation of the top aqueous layer by decantation after centrifugation. Secondly, a final step of passing the DNA through DNA-Easy kit columns was included to obtain good quality DNA for qPCR.

### Real-time PCR

A 222-bp amplicon from *recA *gene of *B. burgdorferi *using RecF and RecR primers and a 154-bp amplicon from mouse *nidogen *gene using NidoF and NidoR primers (Table 1) were amplified by PCR in 0.2 ml optical tubes, as previously described [17], using an ABI7700 sequence detector (Applied Biosystems, NJ). Data was processed using the software from the manufacturer. Amplification was performed in 25 μl reaction mixture containing Amplitaq PCR reaction buffer supplemented with 3 mM MgCl2, 500 ng/μl of bovine serum albumin, 250 μM of each deoxynucleoside triphosphate (dNTP), 0.5 μM of each set of primers and 2.5 U of Amplitaq polymerase. Previous work has shown that a single copy of *recA *and two copies of *nidogen *gene are present per *B. burgdorferi *and mouse genomes respectively [17]. Since genome sizes of *B. burgdorferi *and mouse are 1.5 Mb and 2.5 Gb respectively, 2 ng of *B. burgdorferi *genomic DNA contains approximately 10^6 ^copies of *recA *gene, while 200 ng of mouse genomic DNA contains approximately 10^5 ^copies of *nidogen *gene. For each amplification reaction, 5 μl of the sample was used to minimize the variation due to pipetting error.

### Molecular beacons design

Molecular beacons probes were designed to hybridize to the *recA *and the *nidogen *gene amplicons using the previously described strategies [31]. The lengths of the probe sequences were chosen so that they would form a stable hybrid with the target at 5 to 10°C above the annealing temperature (60°C) of the PCR assay. The 5' and 3' arm sequences of the molecular beacons were designed to form a stable hybrid at 5 to 10°C above the annealing temperature of the PCR assay. After selecting three slightly different probe and arm sequences, the molecular beacon for *recA *amplicon were optimized. These probes were labeled with a Fluorescein (FAM) reporter molecule at their 5' terminals and Black Hole Quencher 1 (BHQ-1) or dabcyl at their 3' terminals. Using similar parameters, a *nidogen *specific molecular beacon was also designed. The Nidogen molecular beacon was labeled with a 5' Tetrachlorofluorescein (TET) reporter molecule and a 3' BHQ-1 quencher. The fluorophores and quenchers were chosen based on the specifications of the spectrofluorometric thermal cycler platform on which the assays were carried out. The sequences of the molecular beacons used in this study are listed in Table 1. A detailed protocol for the synthesis and purification of molecular beacons can be found at http://www.molecular-beacons.org.

PCR products were detected by fluorescence measurement by including SYBR Green I dye (Molecular Probes, OR) or molecular beacons in the assays. The amplification program for SYBR Green I consisted of heating at 95°C for 2 minutes, followed by 50 cycles of heating at 95°C for 15 s, annealing at 60°C for 30 s, polymerization at 72°C for 20 s and fluorescence detection at 80°C for 10 s. Mouse *nidogen *was amplified similarly except that fluorescence was detected at 82°C instead of 80°C. For PCR assays using molecular beacon probes, 200 nM of RecA or Nidogen molecular beacon were included per reaction. The amplification program consisted of heating at 95°C for 2 minutes, followed by 50 cycles of heating at 95°C for 15 s, annealing and fluorescence detection at 60°C for 30 s, and polymerization at 72°C for 20 s.

### Determination of thermal denaturation profiles

In order to determine the melting temperatures of the molecular beacon stem and the molecular beacon probe-target hybrid, a denaturation profile analysis was carried out. For each probe, three tubes containing 200 nM molecular beacon, 3 mM MgCl_2_, 50 mM KCl, and 10 mM Tris-HCl (pH 8.0), in a 50-μl volume were prepared. A two-fold molar excess of an oligonucleotide that is complementary to the molecular beacon probe sequence, a two-fold excess of an oligonucleotide unrelated to the probe sequence, or only buffer were added in these tubes. The fluorescence of each solution was determined as a function of temperature. The thermal cycler was programmed to decrease the temperature of the solutions from 80°C to 30°C in 1°C steps, with each step lasting 1 min, while monitoring fluorescence during each step.

## Authors' contributions

DSS and NP designed and conducted the experiments, SAEM designed molecular beacons and prepared the figures in the manuscript. NP drafted the manuscript. All authors read and approved the final manuscript.
